# Current Strategies to Enhance Recovery following Radical Cystectomy: Single Centre Initial Experience

**DOI:** 10.5402/2012/382843

**Published:** 2012-03-08

**Authors:** Nikhil Vasdev, Praveen L. Pillai, Christopher P. Snowdon, Andrew C. Thorpe

**Affiliations:** ^1^Department of Urology, Freeman Hospital, Newcastle upon Tyne NE7 7DN, UK; ^2^Department of Anaesthetists, Freeman Hospital, Newcastle upon Tyne NE7 7DN, UK

## Abstract

A radical cystectomy (RC) with pelvic lymph node dissection is the gold standard treatment for muscle-invasive bladder carcinoma. The morbidity associated with RC is clearly lower than that in the previous decades; it still continues to remain higher than 30% in the early postoperative period associated with and remains the most effective method for local control. We present current strategies being developed to further enhance recovery in patients undergoing RC and stratifying these into pre, intra, and post operative. We present our current strategies to enhance revcovery in patients undergoing RC which includes a combination of a through preoperative assessment with cardiopulmonary exercise (CPX), preoperative carbohydrate loading drinks, and intraoperative fluid monitoring with the trans-oesophageal Doppler probe (TODP) that may enhance recovery following radical cystectomy. We conclude that using these strategies may not only help in reducing peri/post operative morbidity and the duration of inpatient stay but may also help in enhancing the patient's long-term recovery.

## 1. Introduction

A radical cystectomy (RC) with pelvic lymph node dissection is the gold standard treatment for muscle-invasive bladder carcinoma and remains the most effective method for local control [1]. RC is a major procedure with the potential for serious complications which commonly develop in the early perioperative period [2]. Although the morbidity of RC is clearly lower than that in the previous decades, it still continues to remain higher than 30% in the early postoperative period [1, 2]. RC constitutes major uro-oncological pelvic surgery, and the duration of surgery can range from 3 to 8 hours requiring a significant abdominal incision. The procedure itself is associated with complications, even when carried out by expert surgeons [2]. RC itself generates a very significant proinflammatory response with a high demand for oxygen and tissue perfusion, posing heavy stress on the physiological balance of the patient, and patients are prone to rapid fluid shifts during the intraoperative period.

With an increasing drive to improve and enhance patient recovery during the perioperative period following RC, newer avenues of patient management are being explored in the preoperative workup of patients, preoperative feeding, intraoperative anaesthetic monitoring, and postoperative enhanced recovery. Despite the previous data, the incidence of postoperative ileus continues to be high at 25% and is thought to be the main reason for delayed hospital discharge [3]. Within the United Kingdom, the average length of postoperative hospital stay following RC is between 7 and 14 days postoperatively [4–6].

We review our current methodologies to enhance recovery in patients undergoing an RC. The methodologies are classified into pre, peri, and post operative.

## 2. Our Current Methodologies to Enhance Recovery following RC

### 2.1. Preoperative Methodologies

Major urological surgery promotes a profound physiological reaction mediated by an alteration in catabolic and anabolic hormone balance. The secretion of cortisol and catecholamines leads to an acute-phase response [7, 8] associated with an altered carbohydrate and protein homeostasis leading to a state of hypermetabolism [7, 8]. As a result of this complex physiological stress [9], patients may develop an early stage (i.e., stage 1) of Multi-Organ Dysfunction Syndrome (MODS), which is defined as an increase in fluid volume requirement, mild respiratory alkalosis, oliguria, hyperglycemia, and increased insulin requirements [10, 11]. It is thus important to monitor all patients undergoing an RC thoroughly.

The initiation of the enhanced recovery process commences with timely communication between the patient, urologist, urology stoma nurse specialist, anaesthetist, and primary medical care practitioner. An additional important component in the enhanced recovery process is a preanaesthetic assessment clinic (PAAC).

An important part of the consent process in patients undergoing an RC involves informed decision making (IDM) by the patient. The IDM process helps patients to be fully informed about the potential benefits, risks, alternatives, and recovery paths of an RC. The next step involves a consultation with the urologist where discussions commence involving shared decision making (SDM). Shared decision making and IDM are essential processes to discuss an RC in detail and to ensure patient's decisions match individual values, preferences, expectations, and counselling on urinary diversion. During the SDM process, patients are also told that an RC is a major urological surgical procedure and is associated with a 30% early postoperative period complication rate [1, 2]. Once the patient decides to undergo an RC, they are seen at the PAAC. At our institution, initial assessment at the PAAC is usually performed by a specialist nurse practitioner (SNP). During this process, patients are assessed for their functional capacity. A maximum equivalent activity (MEA) score (Table 1) derived from a structured questionnaire allows an estimate of actual metabolic equivalents (METS) from validated nomograms incorporating MEA and age [12]. This supplements and informs an objective test of functional capacity derived from cardiopulmonary exercise (CPX) testing, which is performed in many patients scheduled to have an RC.

**Figure 1:**
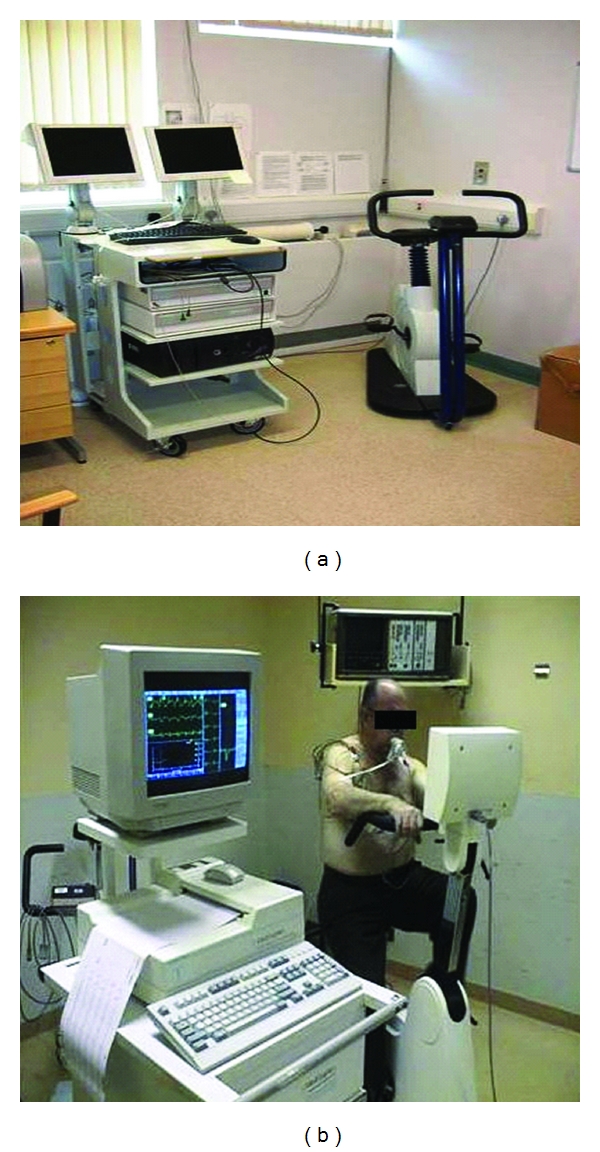
CPX machine (a) and patient performing CPX test (b).

CPX is a noninvasive technique that allows objective evaluation and classification of functional cardiorespiratory reserve based on measurement of oxygen consumption and carbon dioxide production during graded exercise (Figure 1(a)). The test is performed with the patient seated on a bicycle ergometer (Figure 1(b)), breathing through a pressure differential pneumotachograph with respiratory gas analysis and monitored by a 12 lead ECG. The patient is initially asked to take breaths at rest for one minute to establish a baseline. Once the baseline is recorded the patient is then asked to pedal at 60 revolutions per minute. The first three minutes of the test are performed at “zero” watts with no external load on the ergometer flywheel. Analysis of these values is then used to determine various markers of functional cardiorespiratory reserve including maximal (peak) aerobic capacity, the anaerobic threshold (AT) (the point at which anaerobic metabolism supplements aerobic metabolism), oxygen consumption/heart rate ratio (correlate of stroke volume), and the ventilatory equivalents for oxygen and carbon dioxide—VE/VO_2_ and VE/VCO_2_ ratios [13, 14]. Patients identified as having low cardiorespiratory fitness may benefit from the opportunity to improve survival and reduce hospital length of stay through preoperative interventions such as exercise therapy, nutritional intervention or alterations in drug therapy (increase or decrease beta-blockers or statins). Higher risk patients are retained in a postoperative care unit and allow a focus for postoperative outreach services. Low-risk patients have predominantly ward-based care and become an important group for the future development of enhanced recovery programs.

**Figure 2:**
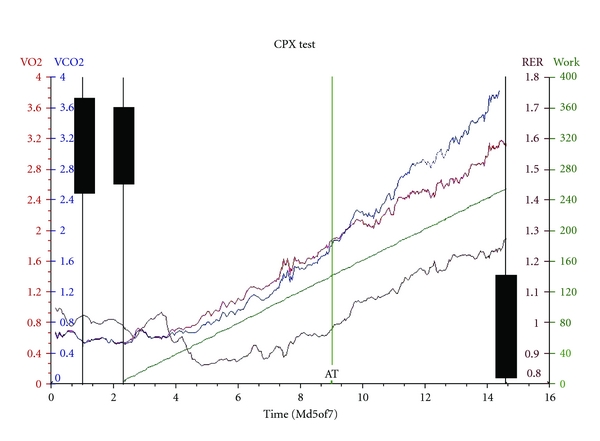
An abnormal CPX trace.

The AT usually expressed in millilitres of oxygen consumed per kilogram body mass per minute time (mL/min/kg) [15] is an important value in determining functional capacity as it is objective and nonvolitional, is independent of the motivation of the patient, and occurs long before the maximum aerobic capacity, without the need for high physical stress [16], which is particularly relevant in the elderly surgical population. A reduction in AT is related to disease and fitness and is only altered at the extremes of age. Studies that have looked at CPX testing as a preoperative screening tool have demonstrated that patients reduced functional capacity have higher mortality and morbidity after major surgery [13, 14, 17]. An abnormal representative CPX trace during exercise is shown in Figure 2.

Upon the completion of the PAAC assessment and outcome of CPX, an estimated risk for the procedure and the appropriate level of postoperative care is defined by the evaluating consultant anaesthetist. This information is used to stratify patients to ward, postanaesthetic care unit (PACU) or high dependency unit (HDU) following RC.

Stoma counselling for our patients commences on the day the patient decides to undergo an RC. Patients are initially seen by urologist and then by our department urology stoma nurse specialist (USNS). Our USNS also provides patients with initial psychological support and organizes a second visit to counsel patients on the implications of the surgery and urinary diversion surgery. At this second visit, the USNS counsels patients using diagrams to illustrate to the patient and their relative the anatomy of the body, that the bladder and prostate will be removed (male) and pelvic clearance for a female. The further repercussions of urinary diversion are also discussed including implications of sexual dysfunction, treatment methods that are available, and should they wish to pursue them once recovered from surgery.

We use a urostomy teaching pack created by ConvaTec—this pack contains an information booklet on the complete journey of having a urostomy and another booklet which is a teaching aid instructing the patient how to apply a model stoma to their abdomen. This allows the patient to visualise their abdomen, apply a pouch (fill it with water if they want to), and then perform everyday tasks, for example, going shopping, driving. It provides the patient with a great deal of confidence and ownership about coping with their stoma postoperatively. The patient would take this pack home with them.

### 2.2. Preoperative Methodologies

All patients undergoing a RC are admitted a day prior to surgery. On admission, patients are examined and instructions from the PAAC are followed through. Efforts can be made to reduce the length of the “nil-by-mouth” period and hence patients are encouraged to eat normally up until six hours before the operation. Clear oral fluids should be allowed until two hours before surgery. Patients receive two sachets of carbohydrate (CHO) loading drinks (PreLoad by Vitaflo, UK) 6 hours apart following admission. The drinks are given up to 2 hours prior to surgery provided that gastric emptying is not impaired. CHO loading drinks are specially formulated oral fluids (complex carbohydrates) for rapid gastric transit due to a relatively low osmolality. CHO loading has been shown to reduce patient anxiety, improve hydration, reduce the body's resistance to insulin and inflammatory response, and improve outcome from surgery at our centre in both urology and colorectal surgery [18]. Patients are seen by the USNS prior to surgery to have their stoma site marked.

### 2.3. Intraoperative Methodologies

During the intraoperative period, accurate monitoring of the patients fluid status is essential to enhance recovery following RC. In our department, we use “individual goal directed patient therapy” using the trans-oesophageal Doppler probe (TODP).

Current strategies for intraoperative fluid balance include a central venous pressure line, arterial line, oxygen saturation monitoring, and continuous pulse and blood pressure recording. In our centre, we use trans-oesophageal Doppler probe (TODP) for an accurate fluid status aimed at recognising/correcting intraoperative hypovolaemia at an early stage. The principal of the TODP originates from the concept of adequate fluid resuscitation to achieve supra-normal cardiovascular function, which has been shown to be associated with an improved outcome by increasing tissue oxygen delivery. This methodology reduces the incidence of gut mucosal hypoperfusion and occult bowel ischemia and therefore also prevents reperfusion injury. A combination of the above factors is responsible for a reduction in the development of multiorgan dysfunction syndrome (MODS) and helps to achieve a reduction in postoperative morbidity and mortality [18].

The concept of TODP aims to enhance patient goal directed therapy (GDT). GDT is the term used to describe the use of cardiac output measurements to guide intravenous or inotropic therapy to ensure adequate tissue perfusion and cellular oxygenation. The rationale behind GDT is to restore and improve oxygen delivery to the tissues and therefore prevent hypoxia.

This TODP is a minimally invasive technique for the assessment and optimisation of fluid balance in patients during an RC. The circulatory data obtained from the TOD is comparable to cardiac output monitoring via a pulmonary artery catheter thermo-dilution method, and the volume status data obtained is also deemed to be more accurate than pulmonary artery wedge pressure estimation [19].

There are 6, 12, and 240 hour Doppler probes available, and the insertion is fairly straightforward through either oral or nasal route. Parameters like age, height, and weight of the patient are required to be entered in the Doppler monitor once the probe is connected, and the machine will calibrate the stroke volume and flow time accordingly. The probe tip is adjusted so that the signals are obtained from the thoracic aortic blood flow (probe is latex free and is 90 cm in length). There are markings at 35, 40, and 45 cm to facilitate correct placement within the oesophagus at approximately T5-6. Descending aortic signals are normally obtained between 35 and 40 cm when probe is placed orally and at 40–45 cm when inserted nasally.

**Figure 3:**
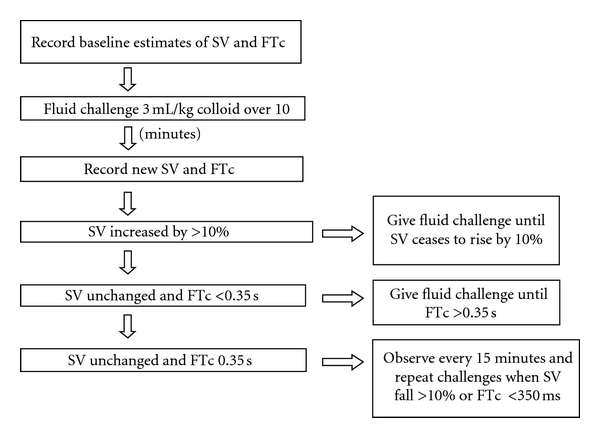
Our intraoperative fluid challenge protocol is being monitored with the trans-oesophageal Doppler probe during radical cystectomy.

Once satisfactory signals are received, which are represented as a waveform in the monitor, stroke volume changes are noted to fluid challenges given with colloid at 3mLs/kg body weight. The oesophageal Doppler probe was inserted under general anaesthesia. Anaesthetic and surgical teams were blinded to the randomisation and additional fluid administration process through screening off both the Doppler monitor (Cardio-Q; Deltex Medical, Chichester, UK) and the researcher who had access to an additional intravenous cannula. The fluid challenges were given until there was no further rise of the stroke volume by 10 percent, which indicates that the left ventricular end diastolic volume is optimised (Figure 3). Continuous circulatory monitoring is performed throughout the operative period, and the fluid challenges are given whenever there is a fall of more than 10 percent of the stroke volume. Thus, an optimisation of intraoperative haemodynamics is achieved that will reduce the gut mucosal hypoperfusion and prevent potential gut mucosal ischemia.

We avoid the placement of nasogastric (NG) tubes in patients undergoing RC. It has now been established that delayed gastric emptying and nausea and vomiting can be induced by an NG tube, and we remove it at the end of an RC unless there is a specific reason to keep it in [20]. Postoperatively NG tubes can affect a patient's ability to mobilise easily and therefore raise a psychological barrier to patients' active participation in their rehabilitation [21].

### 2.4. Postoperative Methodologies

Patients are commenced on free fluids on the day of surgery, and diet is commenced immediately. We also commence patients on two high energy drinks on the first postoperative day. A recent publication from Koupparis et al. [22] recommends the introduction of chewing gum as this causes a faster return of bowel function and leads to a reduced inpatient stay. For the first 24–48 hours patients continue with epidural analgesia to allow sufficient pain control and hence mobilize on day 1 post-RC.

Patients are seen from the day 1 post-RC by our USNS. At this point the USNS will assess the viability of the ileal conduit and ensure that the urostomy appliance is intact and stents are in situ/patent. They encourage the patient to have a look at their stoma at this point. On day 2, the urostomy appliance is used to assess the integrity of the peristomal skin and stoma. From then on daily basis, the patient is encouraged to observe and engage with their stoma care regardless as to how small it may be, for example, removing the pouch. A step-by-step guide is individualised for all patients to encompass their stoma care. This has been very valuable as the patients use it as a prompt. In our experience, most patients are ready and managing their urostomy from day 5 post-RC.

## 3. Discussion

A major development in our unit is the utilization of the TODP to enhance postoperative recovery following RC. Our current fluid protocol (Figure 3) describes the administration of fluids based on TOPD aimed at reducing morbidity secondary to postoperative ileus. This leads to the early establishment of bowel function facilitating earlier hospital discharge in patients undergoing radical cystectomy.

Noblett et al. [18] in a double blind randomized trial studied a group of 108 patients undergoing elective colorectal surgery and randomized them into either standard perioperative fluids (control group) or protocol lead fluid administration based on TODP monitoring (trial group). In the study higher aortic flow time, stroke volume, cardiac output, and cardiac index were seen on the trial group. The study showed a significant reduction in postoperative hospital stay (7 versus 9 days; *P* = 0.005) and a significantly lower intermediate or major postoperative complication rate (2 versus 15 percent; *P* = 0.043). Patients in the trial group also tolerated diet earlier (2 versus 4 days; *P* = 0.029). They were also able to show a significant reduction in the rise of perioperative cytokine interleukin 6 levels in the intervention group.

Conway et al. [23] in a study involving 57 patients undergoing bowel resection used Doppler directed fluid optimization and were able to demonstrate improved cardiac output and reduced admission to critical care. Several orthopaedic studies (Sinclair, Venn) using Doppler directed intraoperative fluid optimization have demonstrated significant reductions in time to fitness for hospital discharge, and hospital stay, although these studies were not randomized or blinded.

At our centre, the second author [24, 25] has conducted an initial evaluation on the effect on the use of the TODP intraoperatively to optimize fluid balance and its effect on enhanced recovery following RC in the trial group after receiving local ethic committee approval. 

The data from this study indicates that fluid monitoring during RC with the TODP improves intraoperative haemodynamics, achieving effective tissue perfusion and reducing physiological stress during surgery. This in turn accelerates postoperative bowel recovery with a reduced incidence of postoperative ileus, allowing better postoperative recovery and early hospital discharge [25]. 

Optimization of intraoperative fluid management has been associated with an optimization of fluid balance within the splanchnic vasculature which further enhances circulatory regulation to the gut mucosa [17, 18, 23, 26, 27]. Indeed one of the initial responses to a sudden decrease in circulatory volume is the redistribution of blood away from the splanchnic bed to vital organs. Even though the pulse rate and blood pressure may remain acceptable during this redistribution, a profound splanchnic hypoperfusion, secondary to hypovolaemia, may ensue. The gut mucosal hypoperfusion which occurs secondary to this will lead to bacterial translocation with endotoxaemia leading to activation of the inflammatory cascades, all of which contribute to the post surgical systemic inflammatory response [17, 26–28]. The aim of fluid intraoperative fluid monitoring using the TODP in patients undergoing RC is to prevent gut ischemia [17, 24–28].

## 4. Conclusion

A combination of a thorough postoperative assessment with cardiopulmonary exercise (CPX), postoperative carbohydrate loading drinks and intraoperative fluid monitoring with the trans-oesophageal Doppler probe (TODP) may enhance recovery following radical cystectomy. These strategies also help in reducing peri/post operative morbidity and the duration of inpatient stay.

## Figures and Tables

**Table 1:** The objective maximum equivalent activity (MEA) score.

Maximum equivalent activity score (1–10)	Clinical activity/equivalent
1	Ability to walking around the house
2	Shortness of breath on eating/dressing
3	Shortness of breath on walking 200 yards flat
4	Shortness of breath on walking 400 yards flat
5	Shortness of breath on walking 1 flights of stairs
6	Shortness of breath on brisk walking
7	Shortness of breath on walking 2 flights of stairs
8	Shortness of breath on walking 3 flights of stairs
9	Shortness of breath on jogging
10	Shortness of breath on brisk swimming

## References

[1] Stein JP, Lieskovsky G, Cote R (2001). Radical cystectomy in the treatment of invasive bladder cancer: long-term results in 1,054 patients. *Journal of Clinical Oncology*.

[2] Chang SS, Cookson MS, Baumgartner RG, Wells N, Smith JA (2002). Analysis of early complications after radical cystectomy: results of a collaborative care pathway. *Journal of Urology*.

[3] Chang SS, Baumgartner RG, Wells N, Cookson MS, Smith JA (2002). Causes of increased hospital stay after radical cystectomy in a clinical pathway setting. *Journal of Urology*.

[4] Frazier HA, Robertson JE, Paulson DF (1992). Complications of radical cystectomy and urinary diversion: a retrospective review of 675 cases in 2 decades. *Journal of Urology*.

[5] Figueroa AJ, Stein JP, Dickinson M (1998). Radical cystectomy for elderly patients with bladder carcinoma: an updated experience with 404 patients. *Cancer*.

[6] Rosario DJ, Becker M, Anderson JB (2000). The changing pattern of mortality and morbidity from radical cystectomy. *BJU International*.

[7] Weissman C (1990). The metabolic response to stress: an overview and update. *Anesthesiology*.

[8] Desborough JP (2000). The stress response to trauma and surgery. *British Journal of Anaesthesia*.

[9] Brodner G, Van Aken H, Hertle L (2001). Multimodal perioperative management—combining thoracic epidural analgesia, forced mobilization, and oral nutrition—reduces hormonal and metabolic stress and improves convalescence after major urologic surgery. *Anesthesia and Analgesia*.

[10] Rosario DJ, Becker M, Anderson JB (2000). The changing pattern of mortality and morbidity from radical cystectomy. *BJU International*.

[11] Maffezzini M, Campodonico F, Canepa G, Gerbi G, Parodi D (2008). Current perioperative management of radical cystectomy with intestinal urinary reconstruction for muscle-invasive bladder cancer and reduction of the incidence of postoperative ileus. *Surgical Oncology*.

[12] Myers JS, Grigsby J, Teel CS, Kramer AM (2009). Nurses’ assessment of rehabilitation potential and prediction of functional status at discharge from inpatient rehabilitation. *International Journal of Rehabilitation Research*.

[13] MacGregor T, Patel N, Blick C, Arya M, Muneer A (2009). Is there a role for cardiopulmonary exercise testing before major urological surgery. *BJU International*.

[14] Older P, Hall A, Hader R (1999). Cardiopulmonary exercise testing as a screening test for perioperative management of major surgery in the elderly. *Chest*.

[15] Older P, Smith R, Courtney P, Hone R (1993). Preoperative evaluation of cardiac failure and ischemia in elderly patients by cardiopulmonary exercise testing. *Chest*.

[16] Weber KT, Janicki JS (1986). *Cardiopulmonary Exercise Testing*.

[17] Arumainayagam N, McGrath J, Jefferson KP, Gillatt DA (2008). Introduction of an enhanced recovery protocol for radical cystectomy. *BJU International*.

[18] Noblett SE, Watson DS, Huong H, Davison B, Hainsworth PJ, Horgan AF (2006). Pre-operative oral carbohydrate loading in colorectal surgery: a randomized controlled trial. *Colorectal Disease*.

[19] Sinclair S, James S, Singer M (1997). Intraoperative intravascular volume optimisation and length of hospital stay after repair of proximal femoral fracture: randomised controlled trial. *British Medical Journal*.

[20] Nelson R, Edwards S, Tse B (2007). Prophylactic nasogastric decompression after abdominal surgery. *Cochrane Database of Systematic Reviews*.

[21] Nelson R, Tse B, Edwards S (2005). Systematic review of prophylactic nasogastric decompression after abdominal operations. *British Journal of Surgery*.

[22] Koupparis A, Dunn J, Gillatt D, Rowe E (2010). Improvement of an enhanced recovery protocol for radical cystectomy. *British Journal of Medical and Surgical Urology*.

[23] Conway DH, Mayall R, Abdul-Latif MS, Gilligan S, Tackaberry C (2002). Randomised controlled trial investigating the influence of intravenous fluid titration using oesophageal Doppler monitoring during bowel surgery. *Anaesthesia*.

[24] Pillai P, Durkan GC, Johnson MI (2010). Reduced physiological stress and improved bowel recovery: achievement in radical cystectomy by intra-operative fluid optimisation using trans-oesophageal Doppler monitoring. *European Urology*.

[25] Pillai P, Durkan G, Johnson M (2009). Optimising intra-operative haemodynamics using oesophageal doppler monitoring in major surgery—does this accelerates bowel recovery?. *Colorectal Disease*.

[26] Kouba EJ, Wallen EM, Pruthi RS (2007). Gum chewing stimulates bowel motility in patients undergoing radical cystectomy with urinary diversion. *Urology*.

[27] Pruthi RS, Chun J, Richman M (2003). Reducing time to oral diet and hospital discharge in patients undergoing radical cystectomy using a perioperative care plan. *Urology*.

[28] Pillai P, McEleavy I, Gaughan M (2011). A double-blind randomized controlled clinical trial to assess the effect of doppler optimized intraoperative fluid management on outcome following radical cystectomy. *Journal of Urology*.

